# Comparison of cementless twin-peg, cemented twin-peg and cemented single-peg femoral component migration after medial unicompartmental knee replacement: a 5-year randomized RSA study

**DOI:** 10.1007/s00402-023-04991-y

**Published:** 2023-08-11

**Authors:** Sebastian Breddam Mosegaard, Anders Odgaard, Frank Madsen, Lone Rømer, Per Wagner Kristensen, Tobias Dahl Vind, Kjeld Søballe, Maiken Stilling

**Affiliations:** 1 Department of Orthopaedic Surgery, Aarhus University Hospital, Palle Juul-Jensens Boulevard 99, 8200 Aarhus N, Denmark; 2 AutoRSA Research Group, Orthopaedic Research Unit, Aarhus University Hospital, Palle Juul-Jensens Boulevard 99, Aarhus N, Denmark; 3 Department of Orthopaedic Surgery, Rigshospitalet, Copenhagen University Hospital, Blegdamsvej 9, 2100 Copenhagen, Denmark; 4 Department of Clinical Medicine, Aarhus University, Palle Juul-Jensens Boulevard 99, 8200 Aarhus N, Denmark; 5 Department of Orthopaedic Surgery, Vejle Hospital, Beriderbakken 4, 7100 Vejle, Denmark; 6 Department of Clinical Medicine, University of Copenhagen, Blegdamsvej 3B, 2200 Copenhagen N, Denmark; 7 Department of Radiology, Aarhus University Hospital, Palle Juul Jensens Boulevard 99, 8200 Aarhus N, Denmark

**Keywords:** RSA, Femoral component migration, Cementless, Cemented, Unicompartmental knee replacement, Bone mineral density

## Abstract

**Background:**

The component design and fixation method of joint arthroplasty may affect component migration and survival. The aim of this study was to compare fixation of cementless twin-peg (CLTP), cemented twin-peg (CTP) and cemented single-peg (CSP) femoral components of medial unicompartmental knee replacement (UKR).

**Methods:**

Eighty patients (mean age = 63 years, 48 males) with medial knee osteoarthritis were randomized in three ways to CLTP (*n = *25), CTP (*n = *26) or CSP (*n = *29) femoral UKR components. The patients were followed 5 years postoperatively with RSA, bone mineral density (BMD), PROMs and radiological evaluation of radiolucent lines (RLL), femoral component flexion angle and complications.

**Results:**

At the 5-year follow-up, femoral component total translation was comparable between the three groups (*p = *0.60). Femoral component internal rotation was 0.50° (95% CI 0.3; 0.69) for the CLTP group, 0.58° (95% CI 0.38; 0.77) for the CTP group and 0.25° (95% CI 0.07; 0.43) for the CSP group (*p = *0.01). BMD decreased peri-prosthetically (range − 11.5%; − 14.0%) until 6-month follow-up and increased toward the 5-year follow-up (range − 3.6%; − 5.8%). BMD change did not correlate with component migration. Lower flexion angle was correlated with higher 5-year subsidence, total translation, varus rotation and maximum total point motion (*p = *0.01). Two patients (1 CLTP, 1 CTP) had RLL in the posterior zone. There were two revisions.

**Conclusion:**

At 5-year follow-up, fixation of UKA femoral components with twin-peg was not superior to the single-peg design. Cementless and cemented twin-peg femoral components had similar fixation. A lower flexion angle was correlated with higher component migration.

**Supplementary Information:**

The online version contains supplementary material available at 10.1007/s00402-023-04991-y.

## Introduction

The use of unicompartmental knee replacement (UKR) for patients with single-compartment medial osteoarthritis in the knee has increased during the past 20 years [1]. The Oxford mobile-bearing UKR has been used for more than 30 years and shows good long-term survival [2, 3]. UKR is often inserted using a minimally invasive surgical technique, and several advantages of UKR over total knee replacement (TKR) have been proposed including shorter recovery time [4], shorter in-hospital stay [1, 5] and better long-term outcomes [6, 7]. Conversely, the national joint registries for England and Wales have shown that UKRs have a higher revision rate than TKRs at 9 years (11.6% vs 3.1%) [8], with one of the most common indications for revision surgery being aseptic loosening [9, 10]. Cemented components have a higher rate of failure due to aseptic loosening (27%) compared with cementless components (4%) [10].

For cemented UKR components, aseptic loosening has been related to insufficient cement mantle with progressive peri-prosthetic radiolucent lines or impingement [11, 12]. In 2007, the cementless Oxford UKR was introduced with an expectancy of avoiding cement-related complications and thereby lowering the revision frequency [5, 8, 13]. Cementless component fixation relies on osseointegration to a surface coating after surgery. Primary mechanical fixation is a prerequisite for lasting fixation of both cemented and cementless components and may depend on several factors such as bone quality, bone cement, surface coating and component design. Furthermore, UKR components are exposed to both axial and rotational stress loading during gait shortly after surgery, and for tibial components, it is well documented that the early migration is associated with increased risk of later aseptic loosening [14, 15]. In 2007, a twin-peg design of Oxford femoral components was introduced with the intention of reducing the internal/external rotational loading stress on the fixation interface.

Radiostereometric analysis (RSA) is a validated and highly accurate method to quantify the migration of prosthesis components that has been proposed as an important instrument in phased introduction of new component designs [16, 17]. The knowledge regarding RSA-measured femoral component migration of TKR and UKR remains limited with the majority of studies reporting results after 2 years [18–21].

The aim of the present study was: (1) to investigate the RSA-measured migration patterns of cementless twin-peg (CLTP), cemented twin-peg (CTP) and cemented single-peg (CSP) femoral components; and (2) to evaluate the potential association between component migration and peri-prosthetic BMD, radiolucent lines (RLL) and femoral component flexion angle.

## Materials and methods

### Participants

**Fig. 1:**
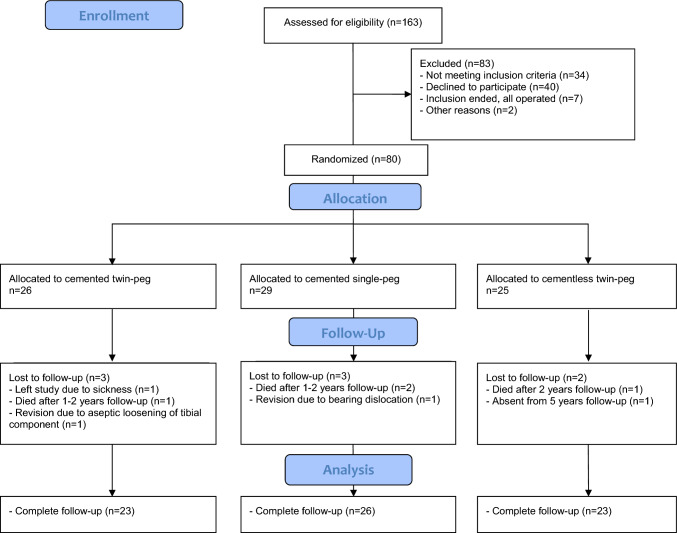
Flow diagram

The present study is a 3-armed single-blinded randomized controlled trial from which data on polyethylene wear have previously been published [13]. Inclusion criteria were painful medial knee osteoarthritis, age > 18 years and informed consent. Please see Horsager et al. for a detailed list of exclusion criteria [13]. A total of 163 patients were assessed for eligibility at two Danish hospitals (Aarhus University Hospital and Vejle Hospital) from June 2009 to October 2011, and 83 patients were excluded (Fig. 1). In the present study, we investigated three randomization arms: CLTP (*n = *25), CTP (*n = *26) and CSP (*n = *29). Patient demographics are presented in Table 1.

**Table 1:** Preoperative patient demographics

	Cemented single-peg *n = *29	Cemented twin-peg *n = *26	Cementless twin-peg *n = *25
Gender, female %	58.6%	32.0%	28.0%
Age, mean (95% CI)	61.7 (58.5; 64.8)	64.3 (60.6; 68.0)	64.6 (60.8; 68.5)
BMI, mean (95% CI)	30.9 (28.7; 33.1)	31.3 (28.9; 33.7)	30.6 (28.9; 32.3)
Radiology			
Component–bone contact, mean (95% CI)	95.6 (92.3; 98.8)	96.9 (94.0; 99.9)	93.9 (86.6; 101.3)
RLL post-op, count	0	1	0
Femoral component flexion angle°, mean (95% CI)	4.5 (2.5; 6.5)	2.0 (− 0.5; 4.4)	4.6 (2.8; 6.3)
KOOS			
Symptoms, mean (95% CI)	57.3 (51.5; 63.0)	59.4 (52.3; 66.5)	53.0 (45.0; 61.0)
Pain, mean (95% CI)	48.1 (43.2; 52.9)	49.6 (44.3; 54.8)	45.2 (39.3; 51.1)
Sport, mean (95% CI)	14.1 (4.4; 23.8)	22.8 (16.4; 29.2)	14.8 (8.6; 21.0)
Activities daily living, mean (95% CI)	56.6 (50.9; 62.3)	57.2 (50.5; 64.0)	49.9 (44.2; 55.6)
Quality of life, mean (95% CI)	31.3 (24.0; 38.5)	36.0 (31.3; 40.7)	30.3 (23.9; 36.6)
SF-36			
Physical functioning, mean (95% CI)	46.9 (39.8; 54.0)	44.8 (38.0; 51.6)	46.0 (38.5; 53.5)
General health, mean (95% CI)	76.0 (69.0; 83.0)	76.8 (70.6; 83.0)	74.6 (67.9; 81.2)
Vitality, mean (95% CI)	49.0 (39.4; 58.6)	56.8 (48.7; 64.9)	60.8 (51.2; 70.4)
Mental health, mean (95% CI)	76.8 (69.9; 83.7)	78.1 (70.6; 85.6)	77.4 (69.7; 85.2)
Role—physical, mean (95% CI)	32.8 (18.5; 47.0)	28.0 (13.5; 42.5)	29.0 (15.3; 42.7)
Bodily pain, mean (95% CI)	40.1 (33.7; 46.6)	39.4 (33.4; 45.5)	38.4 (32.0; 44.9)
Role—emotional, mean (95% CI)	59.8 (43.8; 75.7)	74.7 (59.7; 89.6)	57.3 (40.8; 73.8)
Social functioning, mean (95% CI)	78.0 (69.5; 86.5)	81.0 (71.8; 90.2)	77.5 (68.1; 86.9)

### Sample size

Implant migration between 12 and 24 months > 0.2 mm MTPM is a generally accepted threshold [14]. Using power of 90%, alpha 0.05 and SD 0.2 mm [9], group samples of 22 patients were needed to detect a difference of 0.2 mm between groups, and to account for dropouts, we included 25 patients per group.

### Prostheses

The patients received a phase 3-alpha Oxford medial UKR with ArCom ultra-high molecular weight polyethylene bearing (Zimmer Biomet, Warsaw, IN, USA), and the femoral component was either CLTP, CTP or CSP. The cemented components (both tibial and femoral) were fixed with bone cement (Refobacin Bone Cement R, Zimmer Biomet, Warsaw, IN, USA). The cement was applied to the femoral component concave side and the peg, as well as to the prepared distal femoral bone. CLTP components were inserted by cementless press-fit fixation and had a plasma-sprayed 750-µm-thick titanium coating with an additional plasma-sprayed 55-µm-thick hydroxyapatite (HA) coating.

### Surgical procedure

A standard pre-microplasty surgical procedure as described by the manufacturer was followed for both the cemented and cementless insertions. Prior to the insertion of UKR components, a transparent femoral component template with a stereological point pattern was placed on the sawcut to evaluate the congruency of component–bone area. Lastly, a bead gun (Kulkanon by Wennberg Finmek, Gunilse, Sweden) was used to insert six to eight 1-mm tantalum beads in the peri-prosthetic bone (X-medics, Copenhagen, Denmark). Cement fixation holes were drilled in the femur for improved cement penetration in case of a cemented component, and in the majority of cases when using the cementless component, a few holes were also drilled in the sclerotic subchondral bone with the aim of increasing the number of osteogenic cells at the hydroxyapatite surface.

### Screened radiographs

**Fig. 2:**
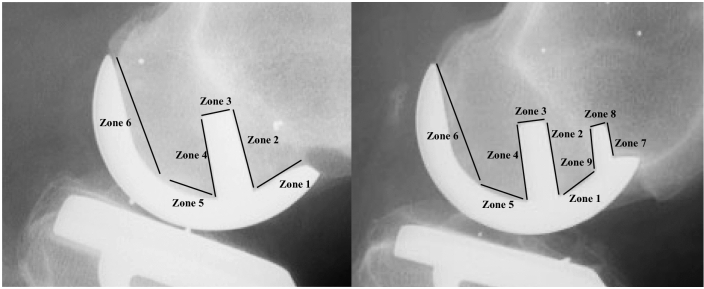
Radiolucent lines zone for single-peg and twin-peg femoral components

**Fig. 3:**
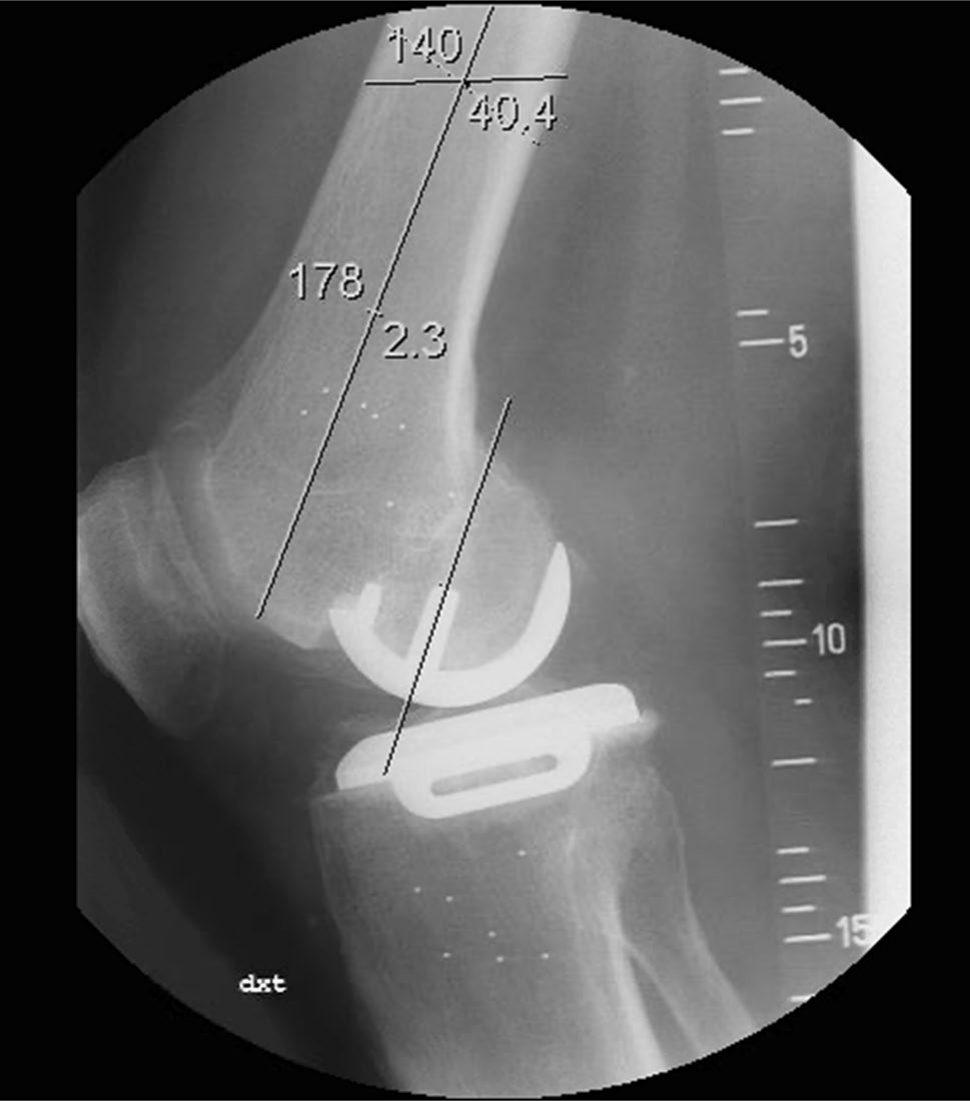
Flexion angle of the femoral component relative to the femoral bone axis measured on X-rays (positive = flexion, negative = extension)

Screened radiographs with focus on an exact lateral view of the femoral component were taken postoperatively and at 1-, 2- and 5-year follow-up and used to evaluate radiolucent lines (RLL) and the potential progression of these. RLL were measured in six zones for the CSP group and in three additional zones around the small peg for the CTP and CLTP groups (Fig. 2). Only radiolucent lines of at least 1 mm in width were counted. Further, the flexion angle of the femoral component (central peg) relative to the central line of the femoral shaft was measured on the postoperative screened radiographs (flexion positive, extension negative) (Fig. 3).

### Radiostereometric analysis (RSA)

Supine stereoradiographs were taken after weight bearing on the first postoperative day and again at 6 weeks, 3 and 6 months, 1, 2 and 5 years postoperatively. To assess the precision, double RSA examinations were performed on all patients at the 6-month follow-up in accordance with the ISO 2013 standards for RSA [22]. After exclusion of two CLTP patients with high condition number (CN > 150) [22], the mean condition number (a measure of the dispersion of the bone markers) in the patient cohort was 59 (95% CI 55; 63), and the mean rigid body error of the bone markers was 0.09 (95% CI 0.08; 0.10).

From 2009 to 2014, the examinations were performed with an Arcoma system (Arco Ceil model 0070-S, Växjö, Sweden). Following 2014, an automated direct digital stereo X-ray system (AdoraRSA suite; NRT, Aarhus, Denmark) was used. The Adora system used wireless CXDI-70C detectors (Canon, Tokyo, Japan), while the Arcoma system used FCR Profect CS detectors (Fujifilm, Vedbæk, Denmark). A uniplanar calibration box (carbon box 24, Leiden, the Netherlands) was used, and the stereographs were analyzed with model-based RSA (version 4.2015, RSAcore, Leiden, the Netherlands) [23]. Implant migration was analyzed with the postoperative stereographs as reference. The same marker model (CN, number of markers) was used throughout analysis for each patient. All migrations are presented for the right leg in a right-handed coordinate system.

Migration of the femoral component is described by translations along the x-axis (+medial/-lateral), y-axis (+subsidence/-liftoff) and z-axis (+anterior/-posterior) and rotations about the x-axis (+flexion/-extension), y-axis (+internal rotation/-external rotation) and z-axis (+varus/-valgus). We used the Pythagorean theorem to calculate total translations (TT) and total rotations (TR) with the formula TT and TR = √(*x*^2^ + *y*^2^ + *z*^2^). The maximum total point motion (MTPM) which represents the vector length of the point of the computer-assisted drawing (CAD) models that migrated the most is also presented.

Finally, we adapted predefined tibial thresholds for continuous migration that was defined as MTPM change from 1 to 2 years ≥ 0.2 mm [14]. For MTPM change from 2 to 5 years, we used modified continuous migration 0.1 mm per year resulting in a continuous migration definition of ≥ 0.3 mm [24]. Double-examination RSA precision was quantified as mean difference, standard deviation difference and coefficient of repeatability (CR) (Table 2).

**Table 2:** RSA double-examination measurement error

Axis	Translations, mm				Rotations, °			
	*x*	*y*	*z*	TT^a^	*x*	*y*	*z*	TR^b^
Mean dif	− 0.059	− 0.005	0.024	− 0.019	0.009	0.169	− 0.242	− 0.033
SD dif	0.258	0.115	0.201	0.139	0.326	0.597	0.765	0.407
CR (± 1.96 * SD dif.)	0.506	0.225	0.394	0.272	0.639	1.170	1.499	0.798

Mean dif. represents the systematic measurement error. SD dif. represents the random variation within the measurement comparing the double examinations. CR (± 1.96 * SD dif.) represents the precision on individual measurements

*CR* coefficient of repeatability; *RSA* radiostereometric analyses; *SD* standard deviation; *TT* total translation; *TR* total rotation

^a^TT was calculated using the Pythagorean theorem (TT = **√**(*x*^2^ + *y*^2^ + *z*^2^))

^b^TR was calculated using the Pythagorean theorem (TR = **√**(*x*^2^ + *y*^2^ + *z*^2^))

### Dual-energy X-ray absorptiometry (DXA)

**Fig. 4:**
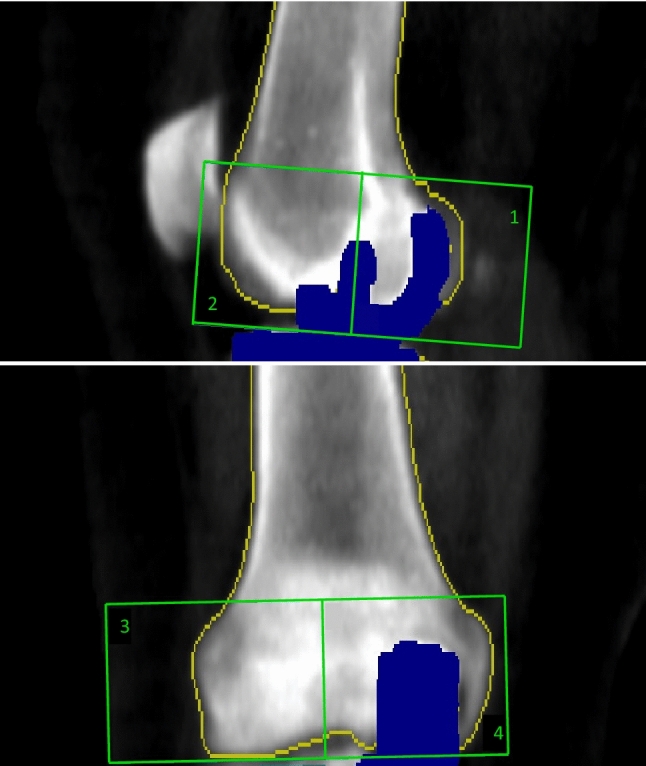
Dual-energy X-ray absorptiometry regions of interest for the evaluation of bone mineral density. The blue area marks the implant and is removed from the BMD measurement. The yellow line marks the bone border for BMD measurement. On LA scans, the division between ROI1 and ROI2 was done in the center of the large peg. On AP scans, the division between ROI3 and ROI4 was done in the intercondylar area

Within three days after surgery, DXA scans of the knee were used to examine the peri-prosthetic BMD of the femoral bone. These scans were used as reference for the evaluation of changes in the follow-up scans at 3 and 6 months, and 1, 2 and 5 years. The BMD was measured in two regions of interest (ROIs) on lateral (LA) scans and two ROIs on the anterior–posterior (AP) scans (Fig. 4). Patient-specific templates of bone edge and ROIs were created on the first scan and used for placement of the ROIs at follow-ups [25]. At 6-month follow-up, double scans were conducted to determine the precision of the scans. These are presented using precision error defined as root mean square standard deviation (g/cm^2^), least significant change as 1.96*√2*RMS SD (g/cm^2^) and finally the percent coefficient of variation [26]. The DXA scans of the knees were performed on a Lunar Prodigy DXA scanner (GE Healthcare, Waukesha, WI, USA) using the “knee scan mode” [25].

### Questionnaires/clinical outcomes

Patients completed the 36-Item Short Form Survey (SF-36) and the Knee Osteoarthritis Outcome Score (KOOS) at baseline, 3 and 6 months, and 1-, 2- and 5-year follow-up. The SF-36 was used to evaluate the patients’ health-related quality of life. It consists of 36 items with eight separate subscales: physical functioning, role limitation (physical), role limitation (mental), bodily pain, general health, mental health, vitality and social functioning [27, 28]. KOOS was used to evaluate the patients’ opinion on their knee and knee problems. It consists of 42 items divided to five separate subscales: pain, symptoms, function in daily living, function in sport and recreation, and knee-related quality of life. Each subscale ranges from 0 to 100 with 100 being the best [29].

### Data analysis and statistics

Normality of continuous data was evaluated using quantile–quantile plots. Depending on the data distribution, continuous group baseline values were compared using one-way analysis of variance or Kruskal–Wallis test depending on the data distribution. Dichotomous data were analyzed using Chi-squared test or test for trend.

Linear mixed models for repeated measurements were used to analyze femoral component migration and BMD change from baseline to 5-year follow-up as dependent variables and cemented single-peg, cemented twin-peg or cementless two-peg as the independent variable. Each patient was used as identifier for within subject variation. The data distribution assumptions were evaluated using model residual quantile–quantile plots and residual vs fitted plots. In case of statistically significant differences among groups, Bonferroni correction for multiple testing was applied. Migration data and BMD change data are presented as predicted means with 95% confidence intervals.

STATA (version 16.1, StataCorp LLC, College Station, TX, USA) was used for statistical analyses. The level of significance was 0.05.

### Ethics

The study was carried out in accordance with the Helsinki II declaration, and all patients gave their informed consent to participate. Approvals were obtained from the local ethics committee (M-20070258; date 15-01-2008), the Data Protection Agency (2008-41-2104 date 28-03-2008) and registered at ClinicalTrials.gov (NCT00679120).

## Results

The CLTP, CTP and CSP groups had comparable baseline demographics, except for gender, with more female patients in the CSP group (58.6%) compared to the CTP group (32.0%) and CLTP group (28.0%) (Table 1). Sensitivity analyses were conducted to evaluate the potential effect of the uneven gender distribution on the analyses, which did not change the outcome of neither the migration analyses nor the BMD analyses.

### Component–bone contact

The percentage of component–bone contact during surgery was similar in the three component groups ranging from 93.9% [95% CI 86.6; 101.3] in the CLTP group to 96.9% [95% CI 94.0; 99.9] in the CTP group (*p > *0.69) (Table 1).

### RSA-measured femoral component migration

**Fig. 5:**
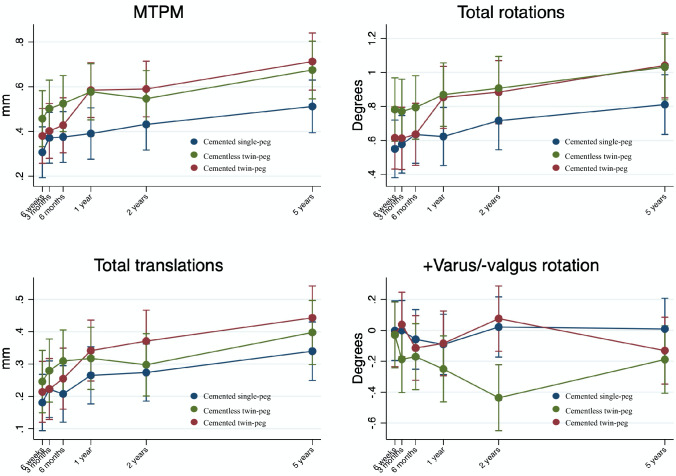
Maximum total point motion (MTPM), total rotations, total translations and varus/valgus rotations of the three femoral component types. Graphs are presented with predicted means from linear mixed models and 95% confidence intervals

The RSA measurement precision is presented in Table 2. Femoral component migrations at 3 and 6 months, and 1, 2 and 5 years are presented in Table 3 and Fig. 5. There was no statistically significant difference in medial/lateral, subsidence/liftoff, anterior/posterior translations or total translations between the three groups at any follow-up. Further, the change in medial/lateral, subsidence/liftoff and anterior/posterior translations from two to 5-year follow-up is comparable among the three groups.

**Table 3:** Migrations of the femoral components as mean (95% CI) along and around the *x*-, *y*- and *z*-axes measured with RSA at 3, 6 months and 1, 2 and 5 years after surgery

Axis	Cemented Single-peg	Cemented Twin-peg	Cementless Twin-peg
Translations, mm			
*x*-axis (+ medial/-lateral)			
6 weeks	0.01 (− 0.06; 0.08)	− 0.01 (− 0.09; 0.07)	− 0.04 (− 0.12; 0.04)
3 months	0.04 (− 0.03; 0.11)	0.00 (− 0.08; 0.07)	− 0.05 (− 0.13; 0.02)
6 months	0.00 ( − 0.07; 0.07)	− 0.02 (− 0.10; 0.06)	− 0.07 (− 0.14; 0.01)
1 year	− 0.04 (− 0.11; 0.03)	− 0.03 (− 0.11; 0.05)	− 0.06 (− 0.14; 0.01)
2 years	− 0.04 (− 0.11; 0.03)	− 0.01 (− 0.09; 0.07)	− 0.09 (− 0.16; − 0.01)
5 years	− 0.09 (− 0.16; − 0.01)	− 0.10 (− 0.18; − 0.02)	− 0.09 (− 0.17; − 0.02)
*y*-axis (+ subsidence/-liftoff)			
6 weeks	− 0.02 (− 0.08; 0.05)	0.01 (− 0.06; 0.08)	0.06 (− 0.01; 0.13)
3 months	0.00 (− 0.06; 0.07)	0.02 (− 0.05; 0.09)	0.08 (0.01; 0.15)
6 months	0.02 (− 0.05; 0.08)	0.03 (− 0.04; 0.10)	0.07 (0.00; 0.14)
1 years	0.04 (− 0.03; 0.11)	0.08 (0.01; 0.15)	0.08 (0.01; 0.15)
2 years	0.04 (− 0.03; 0.10)	0.04 (− 0.03; 0.11)	0.04 (− 0.02; 0.11)
5 years	− 0.01 (− 0.08; 0.06)	0.08 (0.00; 0.15)	0.09 (0.02; 0.16)
*z*-axis (+ anterior/-posterior)			
6 weeks	0.03 (− 0.06; 0.13)	− 0.03 (− 0.13; 0.08)	− 0.04 (− 0.14; 0.07)
3 months	0.05 (− 0.04; 0.15)	0.02 (− 0.08; 0.12)	− 0.01 (− 0.12; 0.09)
6 months	0.06 (− 0.04; 0.16)	− 0.01 (− 0.12; 0.09)	− 0.01 (− 0.11; 0.09)
1 year	0.01 (− 0.08; 0.11)	− 0.04 (− 0.14; 0.06)	0.00 (− 0.10; 0.10)
2 years	− 0.06 (− 0.16; 0.03)	− 0.06 (− 0.17; 0.04)	− 0.02 (− 0.12; 0.08)
5 years	− 0.06 (− 0.15; 0.04)	− 0.19 (− 0.30; − 0.09)	− 0.09 (− 0.19; 0.01)
TT^a^			
6 weeks	0.18 (0.09; 0.27)	0.21 (0.12; 0.31)	0.26 (0.17; 0.35)
3 months	0.22 (0.14; 0.31)	0.22 (0.13; 0.32)	0.29 (0.20; 0.39)
6 months	0.21 (0.12; 0.29)	0.25 (0.16; 0.35)	0.31 (0.21; 0.40)
1 year	0.27 (0.18; 0.35)	0.34 (0.25; 0.44)	0.31 (0.22; 0.40)
2 years	0.27 (0.19; 0.36)	0.37 (0.28; 0.47)	0.30 (0.21; 0.39)
5 years	0.34 (0.25; 0.43)	0.44 (0.35; 0.54)	0.40 (0.31; 0.49)
Rotations, °			
*x*-axis (+ flexion/-extension)			
6 weeks	0.05 (− 0.12; 0.22)	0.04 (− 0.14; 0.23)	0.23 (0.05; 0.41)
3 months	− 0.01 (− 0.18; 0.16)	0.00 (− 0.19; 0.18)	0.21 (0.03; 0.39)
6 months	0.05 (− 0.12; 0.22)	0.09 (− 0.10; 0.27)	0.22 (0.04; 0.40)
1 year	0.06 (− 0.11; 0.24)	0.24 (0.06; 0.43)	0.34 (0.16; 0.52)
2 years	0.05 (− 0.12; 0.22)	0.12 (− 0.06; 0.31)	0.19 (0.01; 0.37)
5 years	0.11 (− 0.07; 0.28)	0.42 (0.23; 0.61)	0.46 (0.27; 0.64)
*y*-axis (+ internal/-external)			
6 weeks	0.06 (− 0.12; 0.23)	0.15 (− 0.04; 0.34)	0.13 (− 0.05; 0.32)
3 months	− 0.06 (− 0.23; 0.11)	0.14 (− 0.05; 0.33)	0.21 (0.02; 0.40)
6 months	0.21 (0.03; 0.38)	0.14 (− 0.05; 0.33)	0.27 (0.08; 0.45)
1 year	0.15 (− 0.03; 0.32)	0.12 (− 0.07; 0.31)	0.28 (0.09; 0.46)
2 years	0.13 (− 0.04; 0.31)	0.09 (− 0.10; 0.28)	0.33 (0.15; 0.52)
5 years	0.25 (0.07; 0.43)	0.58 (0.38; 0.77)	0.50 (0.31; 0.69)
*z*-axis (+ varus/-valgus)			
6 weeks	0.00 (− 0.20; 0.19)	0.03 (− 0.23; 0.18)	− 0.03 (− 0.24; 0.17)
3 months	0.00 (− 0.19; 0.19)	0.04 (− 0.17; 0.25)	− 0.22 (− 0.43; − 0.02)
6 months	− 0.06 (− 0.25; 0.13)	− 0.11 (− 0.32; 0.09)	− 0.21 (− 0.41; − 0.01)
1 year	− 0.09 (− 0.29; 0.10)	− 0.08 (− 0.29; 0.12)	− 0.22 (− 0.43; − 0.02)
2 years	0.02 (− 0.17; 0.22)	0.08 (− 0.13; 0.29)	− 0.42 (− 0.62; − 0.22)
5 years	0.01 (− 0.19; 0.21)	0.13 (− 0.35; 0.08)	− 0.19 (− 0.40; 0.01)
TR^b^			
6 weeks	0.55 (0.38; 0.72)	0.61 (0.43; 0.80)	0.80 (0.62; 0.98)
3 months	0.58 (0.41; 0.75)	0.61 (0.43; 0.80)	0.81 (0.62; 0.99)
6 months	0.63 (0.46; 0.80)	0.64 (0.45; 0.82)	0.83 (0.65; 1.01)
1 year	0.62 (0.45; 0.80)	0.85 (0.67; 1.04)	0.84 (0.66; 1.02)
2 years	0.72 (0.54; 0.89)	0.88 (0.70; 1.07)	0.90 (0.72; 1.08)
5 years	0.81 (0.63; 0.99)	1.04 (0.85; 1.23)	1.07 (0.88; 1.25)
MTPM^c^			
6 weeks	0.31 (0.19; 0.42)	0.38 (0.26; 0.50)	0.48 (0.36; 0.60)
3 months	0.37 (0.26; 0.49)	0.40 (0.28; 0.52)	0.52 (0.40; 0.65)
6 months	0.38 (0.26; 0.49)	0.43 (0.31; 0.55)	0.52 (0.41; 0.65)
1 year	0.39 (0.28; 0.50)	0.58 (0.46; 0.71)	0.57 (0.45; 0.68)
2 years	0.43 (0.32; 0.55)	0.59 (0.47; 0.71)	0.55 (0.43; 0.67)
5 years	0.51 (0.39; 0.63)	0.71 (0.59; 0.84)	0.69 (0.57; 0.81)

^a^Total translation (TT) was calculated using the Pythagorean theorem (TT = √(*x*^2^ + *y*^2^ + *z*^2^)

^b^Total rotation (TR) was calculated using the Pythagorean theorem (TR = √(*x*^2^ + *y*^2^ + *z*^2^)

^c^Maximum Total Point Motion (MTPM)

There was no statistically significant difference in flexion/extension, internal/external, varus/valgus rotation or TR at 6-week and 3-month follow-up among the three groups. Further, there was no statistically significant difference in flexion/extension, internal/external, varus/valgus rotation or TR change from 6-week to 3-month follow-up.

From 2- to 5-year follow-up, the CLTP and the CTP group showed an increased flexion of 0.27° [95% CI 0.07; 0.47] and 0.30° [95% CI 0.10; 0.49], respectively (*p < *0.01). The CSP group remained stable (0.06° [95% CI − 0.12; 0.24]). At 5-year follow-up, there was no difference in flexion/extension between the CLTP and the CTP group. However, the CTP group showed a 0.32° [95% CI 0.06; 0.58] higher flexion than the CSP group at 5-year follow-up (*p = *0.02).

From 2- to 5-year follow-up, only the CTP had a statistically significant increased internal rotation of 0.49° [95% 0.29; 0.69] (*p < *0.01). At 5-year follow-up, the internal/external rotation was comparable in the CLTP and the CTP groups, but the CTP group had 0.33° [95% CI 0.06; 0.60] higher internal rotation than the CSP group (*p = *0.02).

From 2- to 5-year follow-up, the CLTP group had varus rotation of 0.25° [95% CI 0.05; 0.45] (*p = *0.01), whereas the CTP group had valgus rotation of 0.21° [95% CI 0.01; 0.41] (*p = *0.04). The CSP group did not show varus/valgus rotation from 2- to 5-year follow-up. At 5-year follow-up, there was no difference in varus/valgus rotation between the three groups (*p > *0.19). Both the TR change from 2- to 5-year follow-up and the 5-year TR were comparable between the three groups (*p > *0.08).

MTPM was comparable between the CLTP and the CTP groups from 2- to 5-year follow-up and at 5-year follow-up. The CTP group had higher MTPM at 1-, 2- and 5-year follow-up than the CSP group (*p < *0.05). The CLTP group had higher MTPM than the CSP group at 1-year follow-up (*p = *0.03).

There was continuous migration from 1- to 2-year follow-up in 5/23 in the CLTP group, 7/23 in the CTP group and 6/26 patients in the CSP group (*p = *0.76). From 2- to 5-year follow-up, there was continuous migration in 3/23 patients in the CLTP group, 6/23 patients in the CTP group and 3/26 patient in the CSP group (*p = *0.30).

### DXA measured bone mineral density

**Fig. 6:**
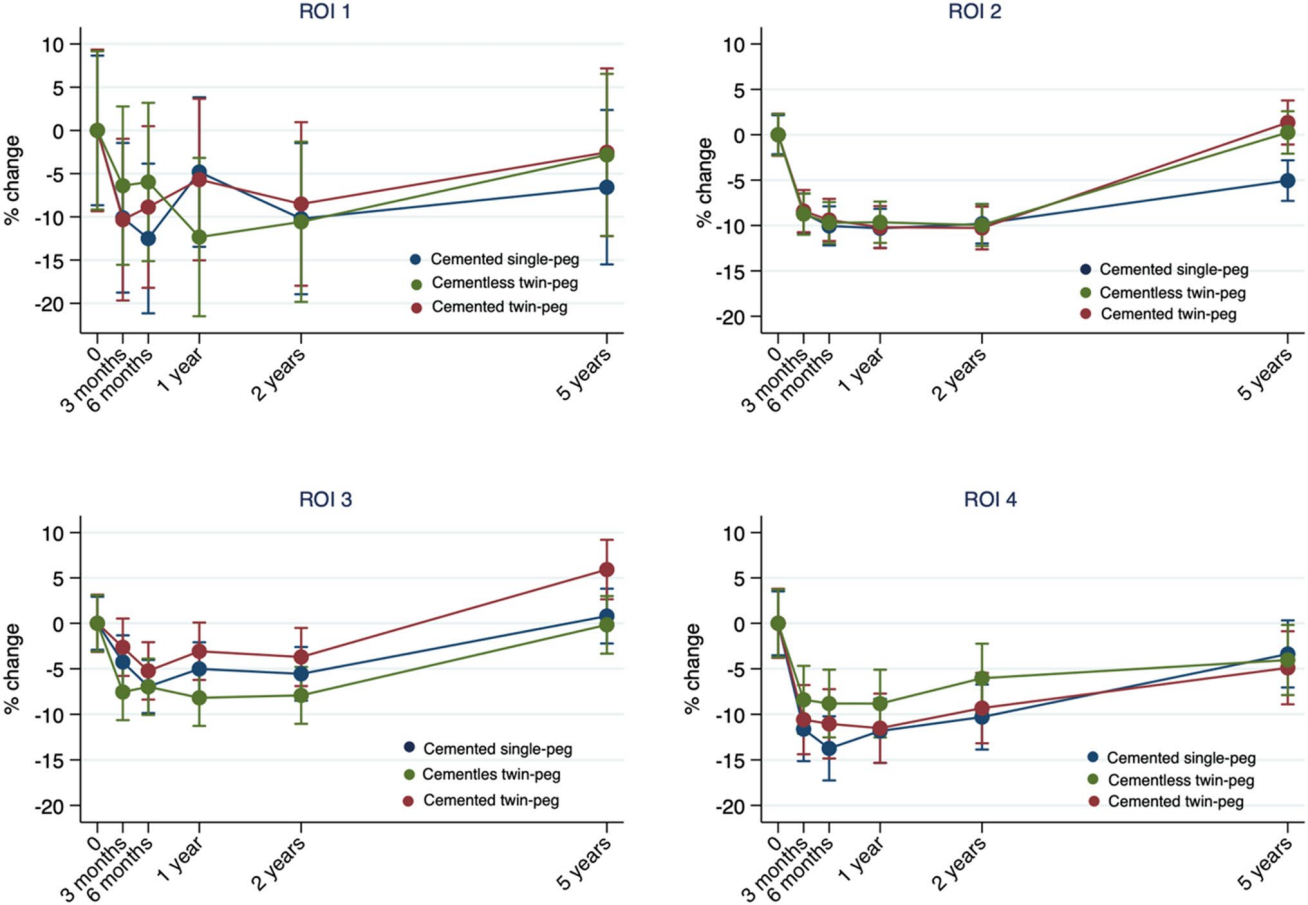
Percentage change of BMD (g/cm^2^) from baseline through 5-year follow-up on LA DXA scans (ROI 1 = posterior and ROI 2 = anterior) and on AP DXA scans (ROI 3 = lateral femoral condyle and ROI 4 = medial femoral condyle)

The precision (CV%) of the BMD measurements was between 16.0% (AP) and 13.9% (LA) in the peri-prosthetic area and between 5.0% (AP) and 2.8% (LA) in the non-prosthetic area (Table 4). There was no statistically significant among-groups difference in baseline absolute BMD (g/cm^2^) in any of the four ROIs (*p > *0.14). BMD changes (%) are presented in (Fig. 6 and a table in electronic supplementary).

**Table 4:** DXA double-examination measurement error

	PE (g/cm^2^)	LSC (g/cm^2^)	%CV (%)
ROI 1	0.2599	0.7205	13.86
ROI 2	0.0460	0.1274	2.75
ROI 3	0.0857	0.2376	5.02
ROI 4	0.1140	0.3160	15.99

The precision was determined using double examination at 6 months. Precision error was calculated using the root mean square standard error (g/cm^2^) and least significant change calculated as 1.96 * √2 * root mean square standard error (g/cm^2^). For comparability reasons, the percentage coefficient of variation (%) was also reported

*DXA* dual-energy X-ray absorptiometry; *PE* precision error; *LSC* least significant change; *%CV* percent coefficient of variation

From postoperative to 3-month follow-up, BMD decreased in the peri-prosthetic as well as the non-prosthetic ROIs on AP and LA scans for both the CSP and CTP groups (*p < *0.02). However, the decrease was not statistically significant in the CLTP group (*p > *0.15). Likewise, at 6 months, the BMD loss on AP scans in the peri-prosthetic area (ROI4) was less pronounced in the CLTP group (*p < *0.04). From 2- to 5-year follow-up, there was a tendency toward a BMD increase in the peri-prosthetic areas on AP and LA scans in all three groups, which was statistically significant for the CSP group (AP, ROI4) (*p < *0.01).

From 2- to 5-year follow-up, the non-prosthetic BMD increased in all three groups on LA scans (ROI2) from group mean 4.6–11.6% and on AP scans (ROI3) from group mean 6.4–9.0% (*p < *0.01). The increase was higher in CTP and CLTP group compared with the CSP group (*p < *0.01). At 5 years, non-prosthetic BMD on LA and AP scans had approximately returned to baseline values for the CTP group (+ 1.4% LA, + 4.9 AP) and the CLTP group (− 0.4% LA, − 0.36% AP), but not on LA scans in the CSP group (− 5.3% LA) (*p < *0.01).

At 5 years, the relative (% change) BMD was similar among groups in ROI1, ROI3 and ROI4. In ROI2, the relative (% change) was lower in the CSP group than the CTP and CLTP group (*p < *0.01).

There was no correlation between femoral component migration (MTPM) and percentage BMD change in any ROI at any follow-up (*p > *0.4).

### Radiolucent lines

No radiolucent lines were present on screened X-rays at any time point in any of the three zones surrounding the small peg in the CLTP and CTP groups. One patient in the CLTP group had a radiolucent line in zone 6 at 1-year follow-up, one patient in the CTP group had a radiolucent line in zone 6 at all follow-ups, and no radiolucent lines were found in the CSP group.

### Component flexion angle

The femoral component flexion angle relative to the femoral bone was not statistically different between the three component groups (*p > *0.15) (Table 1). We found that a lower flexion angle was correlated with higher 5-year subsidence, TT, varus rotation and MTPM (*p = *0.01).

### Questionnaires/clinical outcomes

**Fig. 7:**
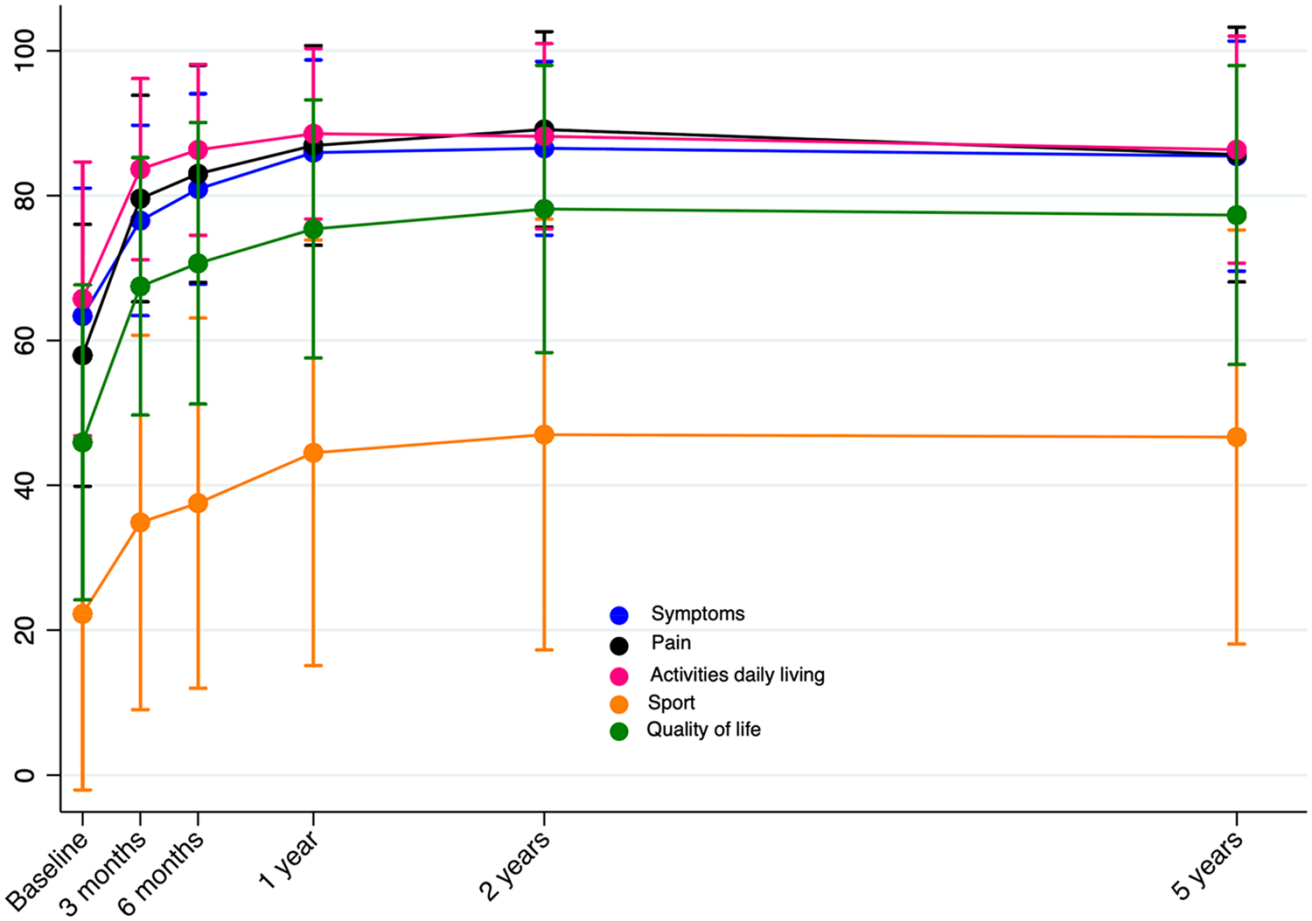
Knee osteoarthritis outcome score subscales for all included patients until five-year follow-up. Presented as means (SD error bars) for the entire patient cohort

**Fig. 8:**
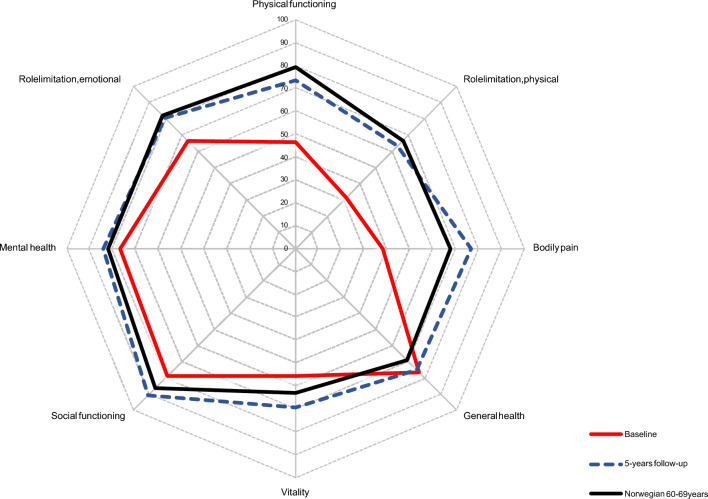
SF-36 subscales at baseline and 5-year follow-up for all patients and a healthy age-comparable Norwegian background population [30]

There was no difference among groups in neither the five KOOS subscales nor the eight SF-36 subscales from baseline to 5-year follow-up. There was an overall improvement in all five KOOS subscales, where most of the improvement happened until 1-year follow-up and remained stable until 5-year follow-up (Fig. 7). Further, patients improved in six of the eight SF-36 subscales from baseline to 5-year follow-up: physical functioning, role limitation (physical), role limitation (emotional), bodily pain, social functioning and vitality. Only the change in general health and mental health was stable from baseline to 5-year follow-up (Fig. 8). Compared to an age-comparable Norwegian background population, patients reached similar or better role limitation (emotional), role limitation (physical), bodily pain, general health, vitality, social functioning and mental health [30]. Only physical functioning did not reach the state of the healthy background population (79.3 points), but did improve from 46.4 points [95% CI 42.0; 50.9] to 73.4 points [95% CI 68.0; 78.8] [30].

### Revisions

No patients in the CLTP group had revision surgery during the 5-year follow-up. One patient in the CTP group was revised to TKR between 2- and 5-year follow-up due to aseptic loosening of the tibial component. One patient in the CSP group was revised to TKR between 2- and 5-year follow-up due to bearing dislocation.

## Discussion

Femoral component stability was hypothesized to improve with a twin-peg component design compared to a single-peg component design. However, this study did not show superior fixation of cemented or cementless twin-peg femoral components over single-peg femoral components.

### RSA-measured femoral component migration

Generally, we found low femoral component migration in all three groups with only TT and TR at 5-year follow-up above the precision limit (CR). This aligns with no revisions of femoral components for aseptic loosening, since the two revisions were related to one tibial component aseptic loosening and one bearing dislocation. Between 2 and 5 years, we did not see improved component fixation for neither the CLTP nor the CTP groups compared with the CSP group as reference. Although the CLTP and CTP femoral components were designed to be inserted at a higher flexion angle relative to the femoral bone, the flexion angle was similar in the three groups. However, we found increased migration in femoral components with a lower flexion angle.

Theoretically, a twin-peg stabilize the UKR femoral component more than a single-peg and a few studies have also indicated this as an advantage. Mohammed et al. conducted a propensity-matched registry-based study comparing a matched group of 2834 patients with Oxford UKR cemented single-peg and 2834 patients with Oxford UKR cemented twin-peg femoral components using data from the National Joint Registry for England, Wales, Northern Ireland and Isle of Man [31]. In this study, they found a statistically significant reduced revision risk in the cemented twin-peg group compared to the cemented single-peg group (hazard ratio = 0.74) and a reduced risk of revision specifically due to femoral component aseptic loosening for the cemented twin-peg group (0.1%) compared to the cemented single-peg group (0.4%). As they were only able to match the patients on data within the registry, other confounders might potentially have affected these results.

Reiner et al. compared the pullout force to failure between cemented single-peg and cemented twin-peg components in 12 human donor cadaver knees, which were randomized to cemented twin-peg in one knee and cemented single-peg in the other knee, and found higher pullout force to failure in the CTP group [32]. It is not explicitly stated whether the prosthesis was pulled out of the cement or whether the cement was pulled out of the bone. In both groups, the cement was applied to the central peg hole and a layer of cement was applied to the surface of the femoral component, with similar amounts of cement (Optipac Refobacin Plus Bone Cement, Biomet Orthopaedics Switzerland GmbH) as applied in the CSP and CTP groups of the present study. The stronger implant–cement fixation could be explained by a larger surface area from an extra peg, but the clinical relevance is questionable as it does not represent a physiological loading scenario. In a different randomized study on human cadaver knees, Reiner et al. found similar component subsidence of Oxford UKR cemented single-peg and cemented twin-peg femoral components using an optical measuring system during cyclical loading at a flexion of 40° and 70° [33]. As expected, a twin-peg does not improve fixation measured as subsidence, which is supported by our clinical results of similar femoral component migration.

Cementless Oxford UKR femoral components are inserted press-fit and the later fixation relies on osseointegration in the hydroxyapatite coating. Campi et al. reported higher RSA-measured migration of Oxford UKR cementless twin-peg than for cemented single-peg femoral components during the first 3 months and thereafter stabilization [34]. Kendrik et al. compared RSA-measured migration of the Oxford UKR cemented single-peg and cementless twin-peg in a randomized trial and found similar femoral component migration a 2-year follow-up in an age-comparable patient cohort with 52% females in the cemented group and 41% females in the cementless group [9]. In both their cemented single-peg and cementless twin-peg groups, they showed slightly higher z-translations (anterior) of 0.22 mm in the cemented single-peg at 2-year follow-up compared to − 0.06 mm in the CS group of the present study. Likewise, z-translations of 0.21 mm for the cementless twin-peg group at 2-year follow-up [9] was higher than − 0.02 in the CLTP group of the present study. The 2-year rotations were generally comparable to the results from our study, with between-study differences of less than 0.19°. However, Kendrik et al. reported 2-year varus/valgus rotation of 0.0° in the cementless twin-peg group, which differs from the 0.42° valgus rotation at 2 years in our CLTP group [9]. The 0.42° valgus rotation is however a small rotation below the CR and SDdif.

Campi et al. presented 5-year migrations of the same patient cohort as in the study by Kendrick et al. [34]. At 5-year follow-up, they found migration results overall comparable to the migration results in the present study. At 5-year follow-up, they found flexion/extension and varus/valgus rotations within 0.2° of the results from our study.

### DXA measured bone mineral density

Peri-prosthetic BMD may influence component fixation and subsequently aseptic loosening [35, 36]. In the present study, all three groups had an initial BMD decrease in the peri-prosthetic and non-prosthetic areas on AP and LA scans, which was less pronounced in the cementless twin-peg group compared with the two cemented groups. This is probably an expression of bone stress shielding in relation to the femoral components and possibly unloading and being less active after surgery. At 5-year follow-up, the BMD was overall comparable between the three groups and roughly normalized to baseline values, which indicate no long-term stress-shielding effect of UKR in the distal femur.

We did not find statistically significant correlations between BMD and component migration.

Tuncer et al. evaluated the femoral BMD after Oxford UKR using cemented and cementless twin-peg femoral components until 2-year follow-up [37]. Combining both cemented and cementless components, they found a mean peri-prosthetic BMD loss of 13% at 1-year follow-up. This is comparable to the 1-year peri-prosthetic BMD loss of 9.4–13.1% in LA scans (ROI1) and of 9.2–11.2% in AP scans (ROI4) of our study. Additionally, Tuncer et al. found the peri-prosthetic femoral BMD to decrease until 6 months and then stabilize or improve [37]. We found that the peri-prosthetic BMD decreased until 6-month to 1-year follow-up and reached the highest postoperative values at 5-year follow-up close to baseline values. A UKR study of seven cemented Duracon unicondylar components (Howmedica International Inc) and 14 cemented Miller-Galante components (Zimmer Biomet) found a femoral BMD decrease at 3-month follow-up ranging from 11.2 to 11.9% and a slightly higher BMD decrease of 15.5% at 1 year compared with our study [36]. The reasons for the difference may lie in demographics, component design, utilized bone cement and analysis method.

### Questionnaires/clinical outcomes

The KOOS and SF-36 improvement was similar among the three groups in our study. Generally, the patients improved their KOOS score beyond the MCID for knee replacement surgeries [38], but they did not reach levels comparable to a healthy background population at any follow-up [39].

We did not identify other studies evaluating KOOS and SF-36 after Oxford UKR, but improvement patterns are very similar to findings for TKA and PFA [40], and yet other studies have evaluated the difference in OKS improvement between Oxford cemented single-peg and cementless twin-peg without showing a difference between groups [9, 34]. Other studies have shown high postoperative patient satisfactions of 97% and 96% with Oxford UKR cemented twin-peg [41, 42]. Panzram et al. conducted a single-center cohort study including the first 30 cementless twin-peg Oxford UKRs in 27 patients and conducted a matched pair analysis with cemented Oxford UKRs [43]. In this study, they found no difference in American Knee Society Score improvement and OKS improvement.

### Radiolucent lines

We only found two patients who each presented with one radiolucent line on the posterior condyle (zone 6). The limited occurrence of radiolucent lines is in accordance with a 5-year study on 512 cementless Oxford UKRs, which only showed radiolucent lines around the tibial component and none around the femoral component [44]. Grabherr et al. studied radiolucent lines in a twin-peg fixed bearing UKR and found radiolucent lines in the posterior zone of 35% of cemented components and in 9% of cementless femoral components (hybrid implant group) with radiolucent lines [45].

### Limitations

Forty patients declined to participate in the study prior to randomization. The baseline characteristics of these patients are unknown, and potential selection bias cannot be ruled out. Eight patients were further lost to follow-up. The baseline characteristics of these patients did not differ from the included patients and therefore we believe that they did not bias the analysis. RSA is a precise and validated method to evaluate component migration patterns, but currently no thresholds for component migration have been established for the femoral components. However, when the migration pattern show stabilization for all patients with very low overall migration measures it can be taken as a positive indication of an expected good long-term fixation. As the observer was able to identify whether the components were cemented/cementless and single-peg/twin-peg, this study is only single-blinded. However, it seems unlikely that this would affect the results as the RSA analyses were conducted using automized RSA analysis software. Although this is a randomized study, the gender distribution was not comparable between the three groups, with relatively more female patients in the CSP group. We conducted sensitivity analyses to explore whether this was related to slightly lower component migrations in the CSP group, which was not found to be the case. Further, the BMD change over time was comparable between the male and the female cases, which leads us to believe that the migration results are not a result of the unbalanced sex distribution.

## Conclusion

After 5-year follow-up, all three femoral component groups showed low femoral component translations and rotations indicating good fixation in all groups, which is emphasized by no midterm revisions for femoral component loosening. Patients generally improved both their KOOS and SF-36 score.

At midterm follow-up, fixation of cemented and cementless twin-peg Oxford UKA femoral components was not superior to the cemented single-peg design. Femoral component fixation had no dependency on peri-prosthetic bone mineral density changes, but lower femoral component flexion angle was correlated with a higher component migration.

### Supplementary Information

Below is the link to the electronic supplementary material.

Supplementary file1 (DOCX 16 KB)
