# Supplementary material for: Comparison of cementless twin-peg, cemented twin-peg and cemented single-peg femoral component migration after medial unicompartmental knee replacement: a 5-year randomized RSA study
Source: Arch Orthop Trauma Surg. 2023 Aug 11;143(12):7169–83. doi: 10.1007/s00402-023-04991-y (PMC10635966; doi:10.1007/s00402-023-04991-y)
Supplement: Supplementary file 1 — Supplementary file1 (DOCX 16 KB) [file 402_2023_4991_MOESM1_ESM.docx]

| Table 5: Mean baseline BMD and follow-up BMD % change at 3 and 6 months, and 1, 2, and 5 years follow-up | | | |
| --- | --- | --- | --- |
|  | Cemented  Single-peg | Cemented  Twin-peg | Cementless  Twin-peg |
| ROI 1 (LA, peri-prosthetic) |  |  |  |
| Baseline, mean g/cm^2^ (95% CI) | 1.48 (1.33; 1.62) | 1.61 (1.46; 1.76) | 1.52 (1.36; 1.62) |
| 3 months, % change (95% CI) | -8.76 (-19.49; 1.97) | -13.71 (-26.27; -1.14) | -6.87 (-14.47; 0.73) |
| 6 months, % change (95% CI) | -11.51 (-21.44; -1.58) | -10.66 (-23.38; 2.07) | -8.30 (-15.99; -0.60) |
| 1 year, % change (95% CI) | -3.99 (-16.23; 8.24) | -9.39 (-21.17; 2.39) | -13.08 (-21.51; -4.65) |
| 2 years, % change (95% CI) | -9.03 (-20.06; 2.00) | -12.0 (-21.60; 2.40) | -12.42 (-23.23; -1.61) |
| 5 years, % change (95% CI) | -5.83 (-16.67; 5.01) | -4.97 (-17.24; 7.31) | -4.68 (-15.78; 6.42) |
| ROI 2 (LA, non-prosthetic) |  |  |  |
| Baseline, mean g/cm^2^ (95% CI) | 1.39 (1.30; 1.47) | 1.52 (1.43; 1.61) | 1.40 (1.30; 1.50) |
| 3 months, % change (95% CI) | -8.83 (-11.32; -6.34) | -8.28 (-11.37; -5.19) | -8.57 (-10.95; -6.19) |
| 6 months, % change (95% CI) | -9.83 (-12.16; -7.49) | -9.46 (-11.94; -6.99) | -10.38 (-12.69; -8.07) |
| 1 year, % change (95% CI) | -10.05 (-12.46; -7.63) | -9.85 (-12.18; -7.52) | -10.15 (-13.28; -7.01) |
| 2 years, % change (95% CI) | -9.66 (-12.14; -7.17) | -9.89 (-12.31; -7.48) | -10.09 (-12.87; -7.30) |
| 5 years, % change (95% CI) | -4.98 (-7.71; -2.26) | +1.39 (-1.67; 4.45) | -0.37 (-3.30; 2.55) |
| ROI 3 (AP, non-prosthetic) |  |  |  |
| Baseline, mean g/cm^2^ (95% CI) | 1.23 (1.15; 1.32) | 1.41 (1.33; 1.48) | 1.38 (1.29; 1.46) |
| 3 months, % change (95% CI) | -3.25 (-6.45; -0.04) | -2.54 (-6.15; 1.07) | -7.24 (-10.12; -4.36) |
| 6 months, % change (95% CI) | -6.69 (-11.24; -2.14) | -5.32 (-8.83; -1.81) | -7.43 (-10.71; -4.15) |
| 1 year, % change (95% CI) | -5.34 (-8.45; -2.22) | -3.48 (-6.78; -0.18) | -8.42 (-11.11; -5.73) |
| 2 years, % change (95% CI) | -5.65 (-9.45; -1.85) | -3.80 (-7.93; 0.32) | -7.93 (-10.95; -4.92) |
| 5 years, % change (95% CI) | +0.79 (-3.18; 4.76) | +4.93 (-0.28; 10.14) | -0.36 (-3.51; 2.79) |
| ROI 4 (AP, peri-prosthetic) |  |  |  |
| Baseline, mean g/cm^2^ (95% CI) | 1.10 (1.02; 1.17) | 1.20 (1.11; 1.29) | 1.11 (1.03; 1.19) |
| 3 months, % change (95% CI) | -12.08 (-14.93; -9.23) | -10.79 (-12.96; -8.61) | -7.61 (-10.92; -4.30) |
| 6 months, % change (95% CI) | -14.04 (-21.35; -6.74) | -11.01 (-13.11; -8.90) | -8.65 (-11.94; -5.37) |
| 1 year, % change (95% CI) | -11.50 (-14.11; -8.90) | -11.23 (-13.63; -8.82) | -9.22 (-12.99; -5.44) |
| 2 years, % change (95% CI) | -10.42 (-13.49; -7.35) | -9.88 (-12.84; -6.93) | -6.19 (-10.39; -2.00) |
| 5 years, % change (95% CI) | -3.59 (-11.69; 4.50) | -5.30 (-8.26; -2.35) | -4.63 (-9.94; 0.68) |
